# Microglia Expressing the Alzheimer's Disease Protective Variant *PLCG2*‐P522R Suppress the NLRP3 Inflammasome

**DOI:** 10.1002/alz70855_100639

**Published:** 2025-12-23

**Authors:** Anuragh Sriram, Matthew Sadgrove, Gwenn A Garden

**Affiliations:** ^1^ University of North Carolina at Chapel Hill, Chapel Hill, NC, USA

## Abstract

**Background:**

GWAS studies demonstrate an association between the P522R variant of the phospholipase C gamma 2 (*PLCG2)* gene and a reduced risk for Alzheimer's disease (AD). In the CNS, PLCG2 is a membrane protein specifically expressed by microglia, the brain‐resident immune cell. Although *PLCG2*‐P522R expresses a protein with mildly enhanced enzymatic activity, the precise mechanism(s) by which protection from AD pathology is achieved remains unclear. Downstream of PLCG2 signaling is the NLRP3 inflammasome, an inflammatory signaling platform in microglia. NLRP3 activity is initiated by sterile, non‐pathogenic stimuli such as ATP or amyloid‐β (Aβ) following specific receptor priming (e.g., LPS, TNFα). Upon activation, microglia assemble an inflammasome complex comprised of apoptosis‐associated speck‐like protein containing CARD (ASC) and mature caspase‐1. This promotes the release of interleukin‐1β (IL‐1β), IL‐18, and the ASC speck. Hyperactivation of the NLRP3 inflammasome in microglia promotes widespread neuroinflammation and Aβ aggregation in AD patients. This study assessed the role of *PLCG2*‐P522R in modulating the NLRP3 inflammasome as a potential mechanism by which the P522R variant protects against AD.

**Methods:**

Cortical microglia were cultured from age‐controlled P3 neonatal mice. Wild‐type (WT), P522R^+/‐^, and P522R^+/+^ microglia were primed with LPS (100 ng/mL) for 3 hours before being triggered with Aβ (5 μM) for 6 hours. ASC speck formation and release were utilized as readouts for inflammasome activation. Specks that were secreted into culture media were isolated and quantified using a novel flow cytometry protocol. Intracellular ASC speck oligomerization was assessed via immunohistochemistry and fluorescent microscopy.

**Results:**

In response to Aβ exposure, P522R^+/+^ microglia formed less and secreted less ASC specks relative to WT microglia. The reduction in speck release was not exhibited by P522R^+/‐^ microglia. Additionally, Aβ exposure alone was able to recapitulate the inflammasome activation profile displayed by WT and P522R microglia treated with LPS + Aβ.

**Conclusion:**

*PLCG2‐*P522R reduces NLRP3 inflammasome activation in microglia. This potentially contributes to the P522R variant's association with decreased AD risk. Furthermore, our novel flow cytometry paradigm for quantifying secreted ASC specks provides a valuable tool for studying inflammasome activity. These results support the development of NLRP3‐targeted therapeutics for AD that mimic the neuroprotective effects of P522R.

